# Supplementary material for: Perceptions of surgeons on surgical antibiotic prophylaxis use at an urban tertiary hospital in Tanzania
Source: PLoS One. 2021 Aug 26;16(8):e0256134. doi: 10.1371/journal.pone.0256134 (PMC8389451; doi:10.1371/journal.pone.0256134)
Supplement: S1 Appendix — (PDF) [file pone.0256134.s001.pdf]

INTERVIEW GUIDE

1. Can you please tell me about yourself and the work you do?
   Probes
   - Age
   - Specialty, super specialty
   - Duration of work/experience
2. What is your understanding of SAP
   Probes
     - Definition
     - Duration
     - Dosage
     - Re-dosing
     - Time of administration
     - Choice
3. Can you please tell me about your experience with Surgical Site Infections (SSI)?
   Probes
   - Patients with SSI
   - What do you attribute to be cause of SSI
   - Previous mitigations due to Surgical Antibiotic Prophylaxis (SAP) and SSI
   - What is the relationship between SAP and SSI
4. How do you come to a decision about which SAP to use?
   Probes
   - Factors influencing decision
     - choice-availability, cost ,pharmaceutical reimbursement
     - dosage- guidelines, weight
     - route-
     - duration- risk of developing SSI, type of wound, previous experience, setting of hospital
   - Patient related factors
   - Colleague related
   - Guidelines
   - Previous experience
5. When should the decision of which SAP to use should be made?
   Probes
   - At time of admission
   - At time of diagnosis
   - During ward rounds
   - In theatre
6. What are your views regarding who should make the decision about which SAP to use?
   Probes
   - Surgeon
   - Resident
   - Junior doctors (interns)
   - Anaesthesiologist
   - Pharmacist
7. What should be done to improve SAP use?
